# Differences in Falls between Older Adult Participants in Group Exercise and Those Who Exercise Alone: A Cross-Sectional Study Using Japan Gerontological Evaluation Study (JAGES) Data

**DOI:** 10.3390/ijerph15071413

**Published:** 2018-07-05

**Authors:** Takahiro Hayashi, Katsunori Kondo, Satoru Kanamori, Taishi Tsuji, Masashige Saito, Akira Ochi, Susumu Ota

**Affiliations:** 1 Department of Rehabilitation and Care, Seijoh University, Tokai, Aichi 476-8588, Japan; ochi@seijoh-u.ac.jp (A.O.); ota-s@seijoh-u.ac.jp (S.O.); 2 Center for Preventive Medical Sciences, Chiba University, Chuo-ku, Chiba 260-8670, Japan; kkondo@chiba-u.jp (K.K.); tsuji.t@chiba-u.jp (T.T.); 3 Center for Gerontology and Social Science, National Center for Geriatrics and Gerontology, Obu, Aichi 74-8511, Japan; 4 Department of Preventive Medicine and Public Health, Tokyo Medical University, Shinjuku-ku, Tokyo 160-8402, Japan; satoru_kanamori@hotmail.com; 5 Human Resource Management Department, ITOCHU Techno-Solutions Corporation, Chiyoda-ku, Tokyo 100-6080, Japan; 6 Department of Social Welfare, Nihon Fukushi University, Mihamacho, Chita-gun, Aichi 470-3295, Japan; masa-s@n-fukushi.ac.jp

**Keywords:** fall prevention, group exercise, cross-sectional study, older adults

## Abstract

This study examined the difference in falls between older adults who participated in group exercise and those who exercised alone. We used cross-sectional data from the Japan Gerontological Evaluation Study. Data were obtained from functionally independent residents aged 65 years or older across 30 municipalities in Japan (*n* = 19,257). Logistic regression analysis was performed with experience of multiple falls over the past year as the dependent variable and type of exercise as the independent variable. Respondents were divided into three groups according to how they performed exercise: (1) non-exercisers (NE, no exercise), (2) those who only exercised alone (IE, individual exercise), and (3) those whose exercise included participation in group exercise (GE, group exercise). In total, 887 (4.6%) respondents reported multiple falls. After adjustment for 10 possible confounders, the GE group had an odds ratio (OR) for falls of 0.75 (95% confidence intervals 0.60–0.95) compared with the IE group. After adjustment for physiological factors and a psychological factor, the OR for the GE group increased slightly; however, an association between falls and exercise type was indicated. Older adults who participate in group exercise may receive additional benefits related to falls prevention compared with those who exercise alone.

## 1. Introduction

Annually, falls occur in more than one-third of persons aged ≥65 years [1,2] and may lead to injury, impaired functioning, and mortality [3,4]. Japan is the country with the highest proportion of older citizens [5], and falls and fractures are among the five main causes of long-term care among older people [6]. Therefore, falls prevention is an urgent public health concern facing Japan and other aging societies.

Previous systematic reviews and meta-analyses of falls prevention have highlighted the importance of increasing physical activity through exercise interventions that improve strength and balance [7,8,9,10]. Previous reports on the efficacy of exercise interventions for falls prevention have noted that it is important to consider the program content and the appropriate amount/frequency of exercise [9,11]. Another aspect of such programs is whether the exercise intervention is performed alone or with others; for example, participation in group activities. The association between exercise interventions and health outcomes may differ by type of exercise (i.e., group exercise or individual exercise) [12,13,14]. A cohort study of Japanese older adults indicated that people who exercised with others had higher subjective health performance than people who exercised alone [12]. Another study involving Japanese older adults reported that sports group participation was associated with a decrease in the likelihood of requiring long-term care when compared with individual exercise [13]. Moreover, a study of Australian adults showed that sports club participants reported more positive benefits for various aspects of quality of life than gymnasium or walking participants [14]. These studies suggest that group exercise has a greater effect on health than exercising alone. Therefore, people who exercise with others may gain additional benefits related to falls prevention than people who exercise alone.

One study found that the risk for falls was approximately 20% lower in people who participated in group exercise more than once a week than in those who did not participate in group exercise, even after controlling for 13 variables [15]. However, the study did not examine the frequency of individual exercise. Therefore, it is unclear whether there is a difference in the risk of falls between older adults who exercise alone and those who participate in group exercise.

This study aimed to examine the difference in falls between older adults who participate in group exercise and those who exercise alone (perform exercise individually). For confirmation, we examined the difference in falls in older adults who exercise alone and those who do not exercise.

## 2. Materials and Methods

### 2.1. Study Sample

We used data drawn from the Japan Gerontological Evaluation Study (JAGES). The JAGES was conducted to examine the social determinants of health among functionally independent older adults aged ≥65 years [16]. Data from the 2013 wave were used in the present study, as these were the most recent data related to our study aim. In the JAGES 2013, respondents were 193,694 older adults across 30 municipalities in Japan. Respondents were selected using complete enumeration (for 13 smaller-scale municipalities) or random sampling (for 17 larger-scale municipalities). Although the selection of municipalities was not random, it included both rural and urban areas and covered most regions of Japan. No participants were eligible for Long-term Care Insurance benefits, which indicated that they had no functional disability.

A mailed, self-administered, questionnaire survey was conducted between October and December 2013, and 137,736 valid responses were obtained (valid response rate: 71.1%). The JAGES questionnaire consists of basic questions to be completed by all respondents, as well as five separate modules that are randomly allocated to participants (20% probability for each module). In this study, we utilized Module D (27,684 respondents), which included items related to individual exercise status. We excluded data from subjects whose sex/age information was inconsistent with the basic resident registration list, on the assumption that there was a risk of distorting the data (i.e., respondents gave incorrect information or a surrogate person completed the responses) (*n* = 1223). We also excluded respondents with missing information on history of falls (*n* = 554), frequency of exercise and participation in group exercise (*n* = 5390). We excluded those who needed assistance in activities of daily living as well (*n* = 1260). The final study sample comprised 19,257 respondents. A lack of information for other question responses was categorized as missing data. Respondents included 9353 men (48.6%) and 9904 women (51.4%), with a mean age of 73.3 ± 6.0 years.

The survey was implemented under a research agreement between each municipality and the JAGES project. The municipalities conducted the survey and provided anonymous data to us, the JAGES researchers. A detailed explanation of the JAGES objectives was sent to potential participants by mail, along with a self-administered questionnaire. JAGES participants were informed that participation in the study was voluntary, and that completing and returning the questionnaire via mail would be considered provision of consent to participate in the study. The ethics committee at Nihon Fukushi University approved the protocol and informed consent procedure for the present study (No. 13–14).

### 2.2. History of Falls

History of falls was assessed with the question, “Have you had any falls in the past year?” Possible answers were “multiple times”, “once”, or “none”. The first category was used as the outcome measure and the latter two categories were combined [15,17]. We defined a “faller” as respondents who reported multiple falls. In previous studies [18,19], fallers were defined as those who had fallen on at least two occasions in the previous 12 months, and non-fallers were defined as those who had not fallen or had fallen only once in the previous 12 months. Moreover, the criteria of more than one self-reported fall in the previous year is used in the Elderly Fall Screening Test, which has both criterion and predictive validity with 83% sensitivity and 69% specificity [20].

### 2.3. Exercising Individually or Group Exercise Participation

Individual exercise was investigated with the question, “How often do you exercise alone?” Those who answered “four or more times a week”, “twice or three times a week”, or “once a week” were classified as “individual exercisers”. Those who answered “once to three times a month”, “a few times a year”, or “never” were not included in this group (i.e., they were not individual exercisers). Participation in group exercise was evaluated with the question, “How often do you participate in a sports group or club?” Those who answered “four or more times a week”, “twice or three times a week”, or “once a week” were classified as “group exercisers”. Those who responded with “once to three times a month”, “a few times a year”, or “never” were not included in this group (i.e., they were not group exercisers). We defined participation in a sports group or club in the community as participation in group exercise.

Based on a previous study [13], respondents were classified into three categories depending on their type of exercise (i.e., group exercise or individual exercise) (Table 1): (1) those who engaged in no individual exercise and no group exercise (NE, no exercise), (2) those who engaged in individual exercise but no group exerciser (IE, individual exercise), and (3) those who engaged in no individual exercise but did engage in group exercise or engaged in both individual exercise and group exercise (GE, group exercise).

### 2.4. Covariates

Parameters used as covariates that may correlate with falls were selected based on previous studies [7,17,21,22,23,24]. These were age, sex, educational attainment, annual-equivalent income, physical ability, instrumental activities of daily of living (IADLs), self-recognition of forgetfulness, current medical conditions related to falls, and number of medications taken. Age was categorized as follows: 65–69, 70–74, 75–79, 80–84, or ≥85 years. Educational attainment was categorized as follows: <6, 6–9, 10–12, or ≥13 years. Annual-equivalent income was calculated by dividing household income by the square root of the number of household members, and was categorized as follows: ≤1,999,999 JPY, 2,000,000–3,999,999 JPY, or ≥4,000,000 JPY. Physical ability was assessed using the two questions (“Do you go upstairs without holding on to the handrail or the wall?” and “Do you get up out of a chair without holding anything?”), and categorized as yes or no. IADLs were assessed using a subscale of the Tokyo Metropolitan Institute of Gerontology Index of Competence (TMIG-IC) questionnaire [25], and categorized as follows: independent (5 points) or non-independent (≤4 points). Self-recognition of forgetfulness was categorized as yes or no. Self-reported current medical treatment for stroke, osteoporosis, joint disease/neuralgia, injury/fracture, impaired vision, and/or impaired hearing was used as a variable for present illness related to falls and categorized into two groups: yes or no. Number of medications taken was categorized as follows: none, <5, or ≥5 medications [24].

Physical activity, depression, social networks, and social support were used to test which aspect of participation in group exercise accounted for falls prevention. Physical activity was assessed by the frequency of vigorous, moderate, and light physical activity in regular daily life, with reference to the English Longitudinal Study of Ageing (Wave 5) [26,27], and categorized as follows: twice a week or more, once a week, less than once a week, or none. Depression was assessed with the short version of the Geriatric Depression Scale-15, which uses a simple “yes” or “no” format and was developed for self-administration in a community setting [28]; responses were categorized as follows: no (≤4 points), mild (5–9 points), or moderate to severe (≥10 points). Reported frequency of meeting friends was used as a measure of social networks, and categorized as follows: four times a week or more, two or three times per week, once a week, several times per month, several times per year, or no meetings. Social support was measured based on four categories (receiving instrumental support, providing instrumental support, receiving emotional support, and providing emotional support). Responses were “yes” or “no”. Those who responded with “yes” to at least one category were classified as having social support.

### 2.5. Statistical Analysis

Logistic regression models were used to calculate the odds ratios (OR) and 95% confidence intervals (CI) for falls. First, to test for group differences, χ^2^ tests were performed on all covariates. Next, we used logistic regression models to determine how falls may be related to whether or not respondents exercised individually or participated in group exercise. Regression analysis was performed with simultaneous forced entry of age, sex, educational attainment, annual-equivalent income, physical ability, IADLs, cognitive impairment (forgetfulness), current medical conditions related to falls, and number of medications taken (Model 1).

Previous studies have shown that the mechanisms underlying health benefits from group exercise (such as participation in sports groups) that are not obtained through individual exercise might involve physiological, psychological, and social factors [29,30,31]. To explain any difference in the relationship between falls and GE, or falls and IE, we added physiological factors (frequency of vigorous, moderate, and light physical activity), a psychological factor (depression), social factors (social networks and social support) to separate models (Model 2 to Model 5), and evaluated the change in the OR associated with group exercise participation. For example, in Model 2 we added “physical activity” to the variables entered in Model 1. Similarly, we added additional variables in subsequent models: depression in Model 3, frequency of meeting friends (a measure of social networks) in Model 4, and social support in Model 5. We used SPSS version 24 (IBM, Armonk, NY, USA) for all analyses, with a 2-tailed significance level set at 5%.

## 3. Results

Table 2 shows respondents’ baseline characteristics. In total, 887 (4.6%) of the 19,257 respondents reported falls in the past year: 6.0% in the NE group, 4.2% in the IE group, and 2.7% in the GE group. The GE group was more likely to report vigorous physical activity once/twice a week or more than the other groups. However, ratios of moderate and light physical activity (either once a week, twice a week, or more) were comparable in the IE and GE groups. The ratio of respondents who were not depressed decreased in the GE, IE, and NE groups, in that order. The same pattern was found for social networks. However, there were similar ratios for social support across the three groups.

Table 3 shows the ORs and 95% CIs for falls associated with each of the three groups. After adjusting for covariates (Model 1) and setting the IE group as the reference, the OR for the GE group was significantly lower at 0.75 (95% CI 0.60–0.95). The OR for the NE group was significantly higher at 1.21 (95% CI 1.04–1.40). 

Physical activity, depression, social networks, and social support were used to test which aspect of participation in group exercise accounted for falls prevention (Table 3: Models 2–5). As mentioned above, when physical activity (Model 2) was added to the covariates in Model 1, the OR for the GE group increased slightly to 0.78 (95% CI 0.61–1.00) (from 0.75, 95% CI 0.60–0.95). The same trend was seen when depression (Model 3) was added to the covariates (from OR 0.75, 95% CI 0.60–0.95 to OR 0.78, 95% CI 0.62–0.98). Addition of either social networks or social support resulted in almost no change in the OR for the GE group.

## 4. Discussion

In this study, we examined falls between older adults who exercised alone and those who participated in group exercise. The main findings were: (1) group exercise participants reported fewer falls compared with those who exercised alone (Table 3, Model 1); and (2) this association remained after adjustment for frequency of physical activity, depression, social networks, and social support (Table 3, Models 2–5).

As highlighted in a previous study [29], participation in group exercise (e.g., participation in sports groups) offered physiological benefits from increased physical activity as well as psychosocial health benefits from social participation. Moreover, previous studies have reported that participation in sports groups reduces the risk of stroke [32] and dementia [33]. A systematic review revealed that there may be greater improvement in psychosocial health benefits through club- and team-based sports than through individual exercise [34]. A single cohort study with Japanese older adults indicated a higher risk of incident functional disability (hazard ratio 1.29, 95% CI 1.02–1.64) among those who did not participate in sports groups compared with those who did participate, even though both groups reported regular exercise [13]. Regarding the relationship between falls and exercise type, the present study showed that the OR for falls was lower in the GE group (Model 1; IE group as the reference). However, we also found that the OR for the NE group was significantly higher than that for the IE group. Previous research indicates that engagement in exercise is important for falls prevention [7,8,9,10]. The present results are consistent with these previous findings.

It may be that the mechanisms underlying health benefits derived from group exercise that are not obtained through individual exercise involve physiological, psychological, and social factors [29,30,31]. The GE group also reported a greater amount of vigorous physical activity, a lower tendency for symptoms of depression, and richer social networks than IE participants. Moreover, physical activity, depression, and social support were associated with falls (Table S1). Therefore, we performed tests to determine if the health protection effects against falls from participation in group exercise could be explained by a higher frequency of physical activity (physiological factor), a lower depression rate (psychosocial factor), and richer social networks and social support (social factors).

The present results showed that when physical activity and depression were added to the covariates in Model 1, the OR of the GE group attenuated slightly. Previous studies on physical activity and social benefits of participation in group activities have highlighted the ability to continue physical activity [35] and the strengthening of social connections [34]. Moreover, in an intervention study that compared psychological effects from group and individual exercise programs for middle-aged and older adults, those who participated in a program comprising both individual and group exercise had higher scores for self-assessment of activities, enjoyment, achievement, satisfaction, and self-recognition than those who only participated in an individual exercise program [36]. It is therefore possible that group exercise participation is more strongly related to physiological and psychosocial factors than individual exercise, and thereby leads to a decrease in falls. However, the changes in ORs were minor, and the effect of group activity showed little change.

Interestingly, when measures of social networks and social support were added to the covariates in Model 1, the OR of the GE group showed no change. A possible reason for this result may be that social networks were not sufficiently evaluated [13], as the frequency of meeting friends was the only measure used. Group differences in the ratio of people with social support were relatively small in this study. In addition, Durbin et al. have reported that social support was not associated with falls [37].

These findings suggest that after adjusting for potential mediators, participation in group exercise was slightly positively correlated with physiological factors and the psychosocial factor. However, the effect of group exercise showed little change. Therefore, it is possible that older adults who participated in group exercise received additional benefits related to falls prevention compared with those who exercised individually. In addition, there may be unmeasured factors such as differences in the characteristics of the exercise program and presence/absence of coaches between the GE and IE groups. Moreover, GE group subjects may also benefit from information exchanges with other subjects on fall prevention tips/strategies. Alternatively, subjects in the GE group may feel more comfortable with their physical capabilities, as they may have experienced fewer falls in the past and are thus are more likely to choose GE rather than more private exercise. Further studies are needed to clarify the health protection effects for falls of participation in group exercise.

The strengths of this study were that it used data from a relatively large sample and controlled for a large number of variables. However, this study had several limitations. First, self-report of falls may not have been accurate [38]. However, the associations between multiple falls and the examined demographic factors (e.g., age, sex, and depression) showed generally expected trends, suggesting that this outcome measure was sufficiently reliable. A prospective study that includes a daily record of falls is desirable. In addition, we did not categorize subjects who fell only once as “fallers”. Second, the influence of differences in respondents’ motor function (e.g., muscle strength and balance ability at baseline) was not controlled. Therefore, we used physical abilities that could be surveyed as part of the JAGES project. Third, the JAGES survey questionnaires were not focused on/framed to measure exercise duration and the content of the exercise programs (balance training, muscle strength training). Therefore, we were unable to consider these factors. Fourth, this study used data from a mailed, self-administered, questionnaire survey. Therefore, we were unable to examine participants’ relationships (including buddy relationships) and detailed social networks. However, this is the first study to use a very large sample to show differences in falls depending on the type of exercise. Fifth, as this was a cross-sectional analysis, we cannot determine causal relationships. In the future, longitudinal research should be conducted to investigate causal relationships.

## 5. Conclusions

The present study shows that older adult group exercise participants have fewer falls compared with those who exercise alone, after adjusting for multiple confounders. This suggests that older adults who participate in group exercise may receive additional benefits related to falls prevention compared with those who exercise alone. Inclusion of physiological and psychosocial factors as covariates slightly attenuated the OR in the GE group. However, the effect of group activity (e.g., participation in sports groups) showed little change. The associations observed in this cross-sectional study should be verified in future longitudinal and interventional studies.

## Figures and Tables

**Table 1:** Three study groups categorized according to frequency of individual exercise and group exercise participation.

		**Participated in Group Exercise**	
		**No Group Exercisers (Less Than Once a week)**	**Group Exercisers (Once a Week or More)**
**Individual Exercise**	**No Individual Exercisers (Less than once a week)**	No Exercise (NE) group	Group Exercise (GE) group
	**Individual Exercisers (Once a week or more)**	Individual Exercise (IE) group	

**Table 2:** Respondents’ baseline characteristics and univariate associations between type of exercise and covariates.

		Total *n* (%)		No Exercise (NE) *n* (%)		Individual Exercise (IE) *n* (%)		Group Exercise (GE) *n* (%)		*p*
*n*		19,257		7598		7849		3810		
Age (years)										
	65–69	5965	(31.0)	2403	(31.6)	2348	(29.9)	1214	(31.9)	<0.001
	70–74	6090	(31.6)	2175	(28.6)	2590	(33.0)	1325	(34.8)	
	75–79	4064	(21.1)	1544	(20.3)	1703	(21.7)	817	(21.4)	
	80–84	2155	(11.2)	949	(12.5)	859	(10.9)	347	(9.1)	
	≥85	983	(5.1)	527	(6.9)	349	(4.4)	107	(2.8)	
Sex										<0.001
	Male	9353	(48.6)	3847	(50.6)	4012	(51.1)	1494	(39.2)	
	Female	9904	(51.4)	3751	(49.4)	3837	(48.9)	2316	(60.8)	
Educational attainment (years)										<0.001
	≥13	4259	(22.1)	1395	(18.4)	1820	(23.2)	1044	(27.4)	
	10–12	7450	(38.7)	2787	(36.7)	3022	(38.5)	1641	(43.1)	
	6–9	7033	(36.5)	3132	(41.2)	2833	(36.1)	1068	(28.0)	
	<6	236	(1.2)	144	(1.9)	76	(1.0)	16	(0.4)	
	missing	279	(1.4)	140	(1.8)	98	(1.2)	41	(1.1)	
Equivalent income (10,000 yen)										<0.001
	High (≥400)	1819	(9.4)	715	(9.4)	685	(8.7)	419	(11.0)	
	Mid (200–399)	6288	(32.7)	2323	(30.6)	2522	(32.1)	1443	(37.9)	
	Low (≤199)	8023	(41.7)	3246	(42.7)	3402	(43.3)	1375	(36.1)	
	missing	3127	(16.2)	1314	(17.3)	1240	(15.8)	573	(15.0)	
Physical ability										
Stand up from the chair without any aids										<0.001
	Yes	16,110	(83.7)	5995	(78.9)	6744	(85.9)	3371	(88.5)	
	No	3027	(15.7)	1544	(20.3)	1063	(13.5)	420	(11.0)	
	missing	120	(0.6)	59	(0.8)	42	(0.5)	19	(0.5)	
Go up stairs without holding rail or wall										<0.001
	Yes	11,626	(60.4)	4144	(54.5)	4986	(63.5)	2496	(65.5)	
	No	7475	(38.8)	3389	(44.6)	2797	(35.6)	1289	(33.8)	
	missing	156	(0.8)	65	(0.9)	66	(0.8)	25	(0.7)	
IADL										<0.001
	Independent	15,707	(81.6)	5691	(74.9)	6595	(84.0)	3421	(89.8)	
	Non-Independent	3288	(17.1)	1772	(23.3)	1159	(14.8)	357	(9.4)	
	missing	262	(1.4)	135	(1.8)	95	(1.2)	32	(0.8)	
Self-recognition of forgetfulness										<0.001
	No	16,252	(84.4)	6201	(81.6)	6736	(85.8)	3315	(87.0)	
	Yes	2867	(14.9)	1347	(17.7)	1048	(13.4)	472	(12.4)	
	missing	138	(0.7)	50	(0.7)	65	(0.8)	23	(0.6)	
Present illness related to falls ^§^										0.001
	No	12,746	(66.2)	5010	(65.9)	5117	(65.2)	2619	(68.7)	
	Yes	6511	(33.8)	2588	(34.1)	2732	(34.8)	1191	(31.3)	
The number of medications										<0.001
	None	4116	(21.4)	1579	(20.8)	1682	(21.4)	855	(22.4)	
	<5	10,918	(56.7)	4051	(53.3)	4482	(57.1)	2385	(62.6)	
	≥5	3934	(20.4)	1821	(24.0)	1579	(20.1)	534	(14.0)	
	missing	289	(1.5)	147	(1.9)	106	(1.4)	36	(0.9)	
Frequency of physical activity										
Vigorous	2 times a week or more	2498	(13.0)	103	(1.4)	825	(10.5)	1570	(41.2)	<0.001
	Once a week	938	(4.9)	51	(0.7)	298	(3.8)	589	(15.5)	
	Less than once a week	1991	(10.3)	632	(8.3)	1004	(12.8)	355	(9.3)	
	None	12,275	(63.7)	6309	(83.0)	4946	(63.0)	1020	(26.8)	
	missing	1555	(8.1)	503	(6.6)	776	(9.9)	276	(7.2)	
Moderate	2 times a week or more	9405	(48.8)	2118	(27.9)	4775	(60.8)	2512	(65.9)	<0.001
	Once a week	1789	(9.3)	512	(6.7)	679	(8.7)	598	(15.7)	
	Less than once a week	2953	(15.3)	1600	(21.1)	1012	(12.9)	341	(9.0)	
	None	4247	(22.1)	2985	(39.3)	1058	(13.5)	204	(5.4)	
	missing	863	(4.5)	383	(5.0)	325	(4.1)	155	(4.1)	
Light	2 times a week or more	12,761	(66.3)	3748	(49.3)	5964	(76.0)	3049	(80.0)	<0.001
	Once a week	1336	(6.9)	502	(6.6)	517	(6.6)	317	(8.3)	
	Less than once a week	1599	(8.3)	955	(12.6)	481	(6.1)	163	(4.3)	
	None	2662	(13.8)	1990	(26.2)	536	(6.8)	136	(3.6)	
	missing	899	(4.7)	403	(5.3)	351	(4.5)	145	(3.8)	
Depression										<0.001
	No	12,414	(64.5)	4414	(58.1)	5222	(66.5)	2778	(72.9)	
	Mild	3113	(16.2)	1503	(19.8)	1208	(15.4)	402	(10.6)	
	Moderate to severe	987	(5.1)	582	(7.7)	324	(4.1)	81	(2.1)	
	missing	2743	(14.2)	1099	(14.5)	1095	(14.0)	549	(14.4)	
Frequency of meeting friends										<0.001
	4 times a week or more	3266	(17.0)	1010	(13.3)	1273	(16.2)	983	(25.8)	
	2 or 3 times per week	3815	(19.8)	1093	(14.4)	1443	(18.4)	1279	(33.6)	
	Once a week	2424	(12.6)	876	(11.5)	1012	(12.9)	536	(14.1)	
	Several times per month	4143	(21.5)	1735	(22.8)	1865	(23.8)	543	(14.3)	
	Several times per year	3683	(19.1)	1755	(23.1)	1576	(20.1)	352	(9.2)	
	None	1525	(7.9)	935	(12.3)	522	(6.7)	68	(1.8)	
	missing	401	(2.1)	194	(2.6)	158	(2.0)	49	(1.3)	
Social support										<0.001
	Yes	18,914	(98.2)	7422	(97.7)	7727	(98.4)	3765	(98.8)	
	No	343	(1.8)	176	(2.3)	122	(1.6)	45	(1.2)	
Fall (*n*, multiple faller)		887	(4.6)	456	(6.0)	327	(4.2)	104	(2.7)	<0.001

IADL: instrumental activities of daily living. **^§^** Stroke, osteoporosis, joint disease/neuralgia, injury/fracture, mental illness, impaired vision, impaired hearing.

**Table 3:** Multivariate adjusted odds ratios and 95% confidence intervals for falls by type of exercise.

	Crude Model		Model 1		Model 2	
	OR (95% CI)	*p*	OR (95% CI)	*p*	OR (95% CI)	*p*
Type of Exercise						
No Exercise (NE)	1.47 (1.27–1.70)	<0.001	1.21 (1.04–1.40)	0.016	1.23 (1.04–1.46)	0.013
Individual Exercise (IE)	1.00 Ref		1.00 Ref		1.00 Ref	
Group Exercise (GE)	0.65 (0.52–0.81)	<0.001	0.75 (0.60–0.95)	0.014	0.78 (0.61–1.00)	0.048
	**Model 3**		**Model 4**		**Model 5**	
	OR (95% CI)	*p*	OR (95% CI)	*p*	OR (95% CI)	*p*
Type of Exercise						
No Exercise (NE)	1.16 (0.99–1.35)	0.062	1.21 (1.04–1.41)	0.014	1.20 (1.03–1.40)	0.019
Individual Exercise (IE)	1.00 Ref		1.00 Ref		1.00 Ref	
Group Exercise (GE)	0.78 (0.62–0.98)	0.030	0.74 (0.59–0.94)	0.011	0.75 (0.60–0.95)	0.015

OR: odds ratio; CI: confidence interval; Ref: reference. Model 1: Crude model + age, sex, educational attainment, equivalent income, physical ability, self-recognition of forgetfulness, present illness related to falls, and number of medications. Model 2: Model 1 + frequency of physical activity. Model 3: Model 1 + depression. Model 4: Model 1 + frequency of meeting friends. Model 5: Model 1 + social support.

## Supplementary Materials

The following are available online at http://www.mdpi.com/1660-4601/15/7/1413/s1, Table S1: Univariate associations between falls and physiological factors, a psychosocial factor, and social factors.
