# Supplementary material for: Differences in Falls between Older Adult Participants in Group Exercise and Those Who Exercise Alone: A Cross-Sectional Study Using Japan Gerontological Evaluation Study (JAGES) Data
Source: Int J Environ Res Public Health. 2018 Jul 5;15(7):1413. doi: 10.3390/ijerph15071413 (PMC6068491; doi:10.3390/ijerph15071413)
Supplement: Supplementary file 1 [file ijerph-15-01413-s001.pdf]

Table S1. Univariate associations of falls with physiological factors, a psychosocial factor, and social factors

		Total	Fallers		OR	95% CI	*p*
		n	*n*	(%)			
Frequency of physical activity							
Vigorous	2 times a week or more	2498	73	(2.9)	1.00	reference	
	Once a week	938	26	(2.8)	0.95	0.60–1.49	0.814
	Less than once a week	1991	63	(3.2)	1.09	0.77–1.53	0.639
	None	12,275	640	(5.2)	1.83	1.43–2.34	<0.001
	Missing	1555	85	(5.5)	1.92	1.40–2.64	<0.001
Moderate	2 times a week or more	9405	386	(4.1)	1.00	reference	
	Once a week	1789	57	(3.2)	0.77	0.58–1.02	0.069
	Less than once a week	2953	119	(4.0)	0.98	0.80–1.21	0.859
	None	4247	280	(6.6)	1.65	1.41–1.93	<0.001
	Missing	863	45	(5.2)	1.29	0.94–1.76	0.121
Light	2 times a week or more	12,761	506	(4.0)	1.00	reference	
	Once a week	1336	74	(5.5)	1.42	1.11–1.82	0.006
	Less than once a week	1599	79	(4.9)	1.26	0.99–1.61	0.063
	None	2662	171	(6.4)	1.66	1.39–1.99	<0.001
	Missing	899	57	(6.3)	1.64	1.24–2.17	0.001
Depression							
	No	12,414	403	(3.2)	1.00	reference	
	Mild	3113	239	(7.7)	2.48	2.10–2.92	<0.001
	Moderate to severe	987	106	(10.7)	3.59	2.86–4.49	<0.001
	Missing	2743	139	(5.1)	1.59	1.31–1.94	<0.001
Frequency of meeting friends							
	Four or more times a week	3266	153	(17.2)	1.00	reference	
	Two/three times per week	3815	146	(16.5)	0.81	0.64–1.02	0.074
	Once a week	2424	110	(12.4)	0.97	0.75–1.24	0.794
	Several times per month	4143	172	(19.4)	0.88	0.71–1.10	0.266
	Several times per year	3683	187	(21.1)	1.09	0.87–1.35	0.449
	None	1525	94	(10.6)	1.34	1.03–1.74	0.032
	Missing	401	25	(2.8)	1.35	0.87–2.09	0.174
Social support							
	Yes	18,914	860	(4.5)	1.00	reference	
	No	343	27	(7.9)	1.79	1.20–2.67	0.004

OR, odds ratio; CI confidence interval.
